# Effect of Sodium Hypochlorite 0.05% on MMP-9 Extracellular Release in Chronic Wounds

**DOI:** 10.3390/jcm12093189

**Published:** 2023-04-28

**Authors:** Rosanna Maniscalco, Giorgina Mangano, Alessandra Capezzone de Joannon, Matteo Vergassola, Sara Zucchi, Ferdinando Mannello, Lorella Ragni

**Affiliations:** 1 Unit of Clinical Biochemistry, Section of Biochemistry and Biotechnology, Department of Biomolecular Sciences, University of Urbino Carlo Bo, 61029 Urbino, Italy; 2 Scientific Consultant, ToxHub S.r.l. Albano Laziale, 00073 Rome, Italy; 3 Global R&D Angelini Pharma S.p.A, Via Vecchia del Pinocchio, 22, 60131 Ancona, Italy

**Keywords:** sodium hypochlorite, matrix metalloproteinases inhibition, gelatinolytic activity, chronic wound

## Abstract

Background: In chronic wounds, high concentrations of matrix metalloproteinases (MMPs) can cause excessive proteolysis and slow wound healing. Consequently, restoring a proper MMP balance can help reduce the risk of a chronic wound. An antiseptic solution containing 0.05% sodium hypochlorite (Amukine Med 0.05%, Angelini S.p.A.; hereafter termed NaClO solution) is available on the market. The NaClO solution was proven effective and safe in managing infected skin wounds. To further characterize its activity, this study evaluated the in vitro activity of the NaClO solution on the monocyte release of MMPs. Methods: Human monocytic THP-1 (ATCC^®^ TIB-202™) cell lines were differentiated into macrophages and treated with different concentrations of NaClO (from 0.05% to 5 × 10^−7^%). In addition, the THP-1 cell line was stimulated with wound fluid (WF) from patients with active venous leg ulcers in the inflammatory phase. The effect of NaClO (0.025–0.0062%) was also evaluated on healthy human peripheral blood serum samples. The effects of treatments on the gelatinolytic activity of MMP-9 were evaluated by gelatin zymography. The effects on MMPs release were evaluated through the Pro™ Human MMP 9-plex Assay. An exploratory scratch wound healing assay was also performed. Results: The NaClO solution reduced the gelatinolytic activity of MMP-9 and its activated form. The downregulation of MMP-9 gelatinolytic activity was also observed in peripheral blood serum. The MMPs profile showed a reduction in MMP-1 release (*p* < 0.05) and a slight reduction of the release of MMP-9 and MMP-12 after the treatment with LPS and the NaClO solution. A slight improvement in wound healing was observed after macrophage activation and treatment with the NaClO solution. Conclusions: The results obtained suggest a possible ability of the NaClO solution to modulate the proteolytic pathways in the wound microenvironment, further characterizing its activity and use in clinical practice during wound care.

## 1. Introduction

Wound healing is a cascade of ordinate and partially overlapping events involving different cell populations with precise functions [1]. Among them, matrix metalloproteinases (MMPs) play a crucial role [2]. Twenty-four different MMPs have been described, with varying substrate specificities and multiple activities [3,4]. Within the wound healing process, their main function is to degrade the damaged extracellular matrix (ECM) during the inflammatory phase, to rupture the capillary basement membrane for angiogenesis and cell migration during the proliferation phase, and to contract and remodel tissue in the remodeling phase [5,6]. In chronic wounds, where the orderly healing process is compromised, abnormal levels of MMPs have been related to altered healing [1,7,8]. Indeed, high concentrations of MMPs can cause excessive proteolysis, tissue degradation, and slow wound healing. Among the various MMPs, MMP-2 (gelatinase A) and MMP-9 (gelatinase B) have been found to be increased in chronic wound tissues and exudates compared with acute wounds and adjacent normal skin [1,9,10,11]. Consequently, the definition of interventions able to restore a proper MMP balance can help reduce the risk of progression to a chronic wound.

Solutions containing sodium hypochlorite can be considered among the first choice in options for skin wound care [12]. Sodium hypochlorite has proven effective against a wide range of microorganisms, as well as exerting an anti-inflammatory action in the absence of any irritant or cytotoxic reaction and being useful in biofilm removal [13,14]. Moreover, its activity over MMPs has been described [15,16].

A 0.05% sodium hypochlorite-based solution (Amukine Med 0.05%, Angelini S.p.A.; hereafter termed NaClO solution) is available on the market. The NaClO solution is an antiseptic, isotonic (0.9%) solution at pH 9.5 characterized by a high degree of purity, stability, and biocompatibility. Its use is indicated for the disinfection and cleaning of injured skin and to support the wound healing process. According to the most recent regulations and guidelines in the field of wound healing, in the NaClO solution, the sodium tetraborate decahydrate, in consideration of a possible reduction in its use, was replaced with pharmaceutical-grade sodium bicarbonate, and the chemical-grade sodium hydroxide was replaced with pharmaceutical-grade soda to increase the purity of the formulation [13,17].

The NaClO solution was proven effective and safe in managing infected skin wounds [13]. To further characterize its activity, this study aimed to evaluate the in vitro activity of the NaClO solution on the monocyte release of MMPs, with a particular focus on MMP-9. Within this study’s scope, the activity of the NaClO solution was also evaluated on human peripheral blood serum samples.

## 2. Materials and Methods

### 2.1. Cell Culture and Treatments

Human monocytic THP-1 (ATCC^®^ TIB-202™) cell line (American Type Culture Collection; Manassas, VA, USA) was grown in standard culture conditions, and experimental procedures were performed with serum-free media to avoid the recovery of endogenous bovine serum MMPs. THP-1 cells were differentiated into macrophages by adding 50 ng/mL Phorbol-Myristate acetate (PMA) for 24 h. Macrophages were treated with different concentrations of NaClO (from 0.05% to 5 × 10^−7^%). Treatments were added for 30 min to 6 h, in the presence or absence of 10 μg/mL of lipopolysaccharide from *Escherichia coli* (LPS); afterward, culture media were discarded, and cells were maintained at 37 °C, 5% CO_2_ in fresh serum-free medium up to 24 h from the addition of treatments. Additionally, the THP-1 cell line was stimulated with wound fluid (WF) from patients with active venous leg ulcers in the inflammatory phase, as previously described [18], and treated with NaClO (0.025%) for 1 h. The effect of NaClO (0.025–0.0062%) was also evaluated in the same conditions on samples of human peripheral blood serum from healthy subjects. Activation of zymogens was achieved by incubating supernatants with 2 mM p-amino phenyl-mercuric acetate (APMA). Activation assay (2 mM APMA, 6 h, 37 °C) was performed after the treatment with the NaClO solution (0.025–0.0031%) for 1 h. Cell viability was assessed by Trypan blue exclusion test. The LUNA-II™ Automated Cell Counter (Logos Biosystems, Gyeonggi-do, Republic of Korea) was used to quantify the number of total, viable, and dead cells in each sample. The cytotoxicity of the differentiating (PMA, WFs) and stimulating (LPS, APMA) treatments and of the NaClO solution was evaluated by MTT assay. Each experiment on the serum-free conditioned medium was performed in triplicate in at least two independent experiments.

### 2.2. Zymography

Effects of treatments on the gelatinolytic activity of MMP-9 were evaluated in serum-free culture media after 1 h of NaClO treatment followed by 24 h of fresh medium replacement (post-incubation period) by gelatin zymography as previously described [19]. Briefly, gelatin zymography was carried out both with fixed-concentration (7.5%) polyacrylamide separating gels copolymerized with 3 g/L 90 Bloom Type A gelatin. Separating and stacking gels were based on the standard Laemmli method. Zymogram gels were run using a Bio-Rad Mini-Protean Tetra Cell apparatus (Bio-Rad, Segrate, Italy) in cold SDS running buffer (25 mM Tris, 192 mM glycine, and 0.1% *w*/*v* SDS) at a constant voltage of 120 V, for about 2 h. After electrophoresis, gels were washed twice (20 + 20 min) at room temperature on a rotary shaker in Triton X-100 2.5% to gently remove SDS. The gels were washed with distilled water and incubated for 22 h at 37 °C in an enzyme incubation buffer (EIB) containing 50 mM Tris, 5 mM CaCl_2_, 100 mM NaCl, 1 mM ZnCl_2_, 0.3 mM NaN_3_, 0.2 g/L of Brij^®^-35, and 2.5% *v*/*v* of Triton X-100, pH 7.6. Staining was performed using Coomassie Brilliant Blue R-250 (0.2% *w*/*v* Coomassie in 50% *v*/*v* methanol and 20% *v*/*v* acetic acid). Gels were maintained in destaining solution (50% *v*/*v* methanol and 20% *v*/*v* acetic acid) until clear gelatinolytic bands appeared against the uniform dark-blue background of undigested protein substrate. After gel scan acquisition, bands were measured densitometrically with the LabImage 1D image analyzer (Kapelan, Leipzig, Germany).

### 2.3. Magnetic Multiplex Immunoassay

Effects of NaClO treatment (0.025%, 1 h of treatment) on the release of MMPs involved in inflammation and wound healing pathways were evaluated after 24 h post incubation through the Pro™ Human MMP 9-plex Assay (including MMP-1, MMP-2, MMP-3, MMP-7, MMP-8, MMP-9, MMP-10, MMP-12, MMP-13), as previously described [18]. The protein concentrations (expressed as pg/mL) were calculated through a standard curve. Data obtained in the different treatments are expressed as % change vs. control (RPMI 1640 medium).

### 2.4. Scratch Wound Healing Assay

THP-1 differentiated into macrophages were grown in a complete growth medium to confluence in 24-well plates and then serum starved (to induce quiescence) for 24 h in a serum-free medium before treatment. The cell monolayer was then subjected to two scratches on a pre-drawn cross under the plate as a reference point. Cells were washed twice with PBS and incubated in 10 μg/mL LPS with or without 0.025% NaClO for 1 h, followed by a 24 h post-incubation time. The wound surface area was quantified immediately following wounding and after 8 h and 24 h. Phase-contrast images were acquired on a Nikon Eclipse TS100 microscope. All assays were performed in triplicate.

### 2.5. Statistical Analysis

Statistical analyses were determined by nonparametric one-way ANOVA followed by a post hoc test, according to variable characteristics, using Prism 5 software (GraphPad, Boston, MA, USA); *p* < 0.05 was considered statistically significant.

## 3. Results

### 3.1. Cytotoxicity Assay

**Figure 1:**
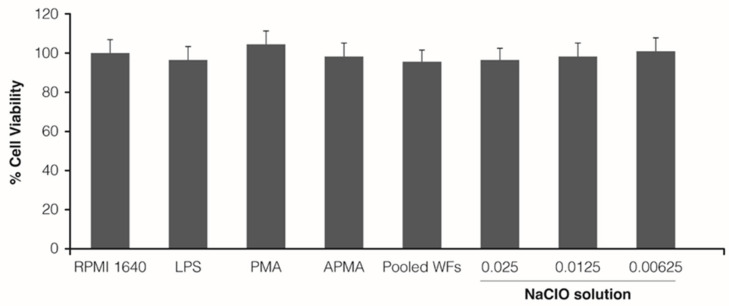
Percentage of viable THP-1 monocytes after the treatment with LPS, PMA, APMA, pooled WFs, or different concentrations of the NaClO solution (0.025, 0.0125, and 0.00625%). Mean ± SD are represented.

The percentage of viable cells detected after the treatment with differentiating and stimulating factors and with different concentrations of the NaClO solution was comparable to the control condition (RMPI 1640 medium) (Figure 1).

### 3.2. Zymography

#### 3.2.1. Differentiated Macrophages

**Figure 2:**
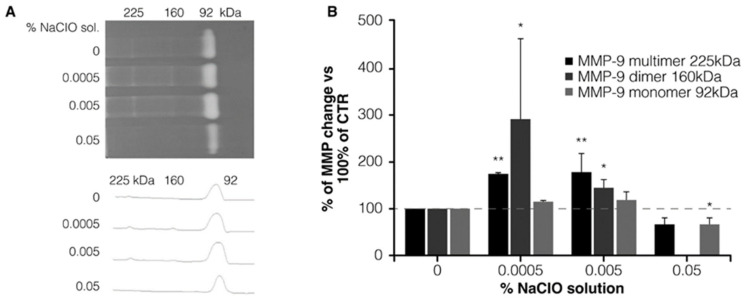
Zymography. (**A**) Gelatinolytic bands at 225, 160, and 92 KDa related to the gelatinolytic activity of MMP-9 in the form of multimer, dimer, and monomer, respectively; (**B**) Percentage of MMP change after the treatment with 0.0005, 0.005, and 0.05% NaClO compared with the control condition (* *p* < 0.05; ** *p* < 0.005).

Zymographic analysis of serum-free media of differentiated macrophages showed the presence of three gelatinolytic bands at 225, 160, and 92 KDa related to the gelatinolytic activity of MMP-9 in the form of multimer, dimer, and monomer, respectively (Figure 2A).

The treatment with 0.0005 and 0.005% NaClO solution was associated with a significant increase in the gelatinolytic activity of MMP-9 at 225 kDa and 160 kDa (percentage of increase vs. 100% of CTR: 172.5 ± 6.25 and 177.5 ± 39.6, *p* = 0.001 and *p* = 0.01, respectively; Figure 2B), compared with untreated controls. On the other hand, a significant decrease in the monomeric form of MMP-9 (92 kDa) was observed after the treatment with the NaClO solution (0.05%; percentage of increase vs. 100% of CTR: 62.35 ± 16.85, *p* < 0.05, as shown in Figure 2B).

Furthermore, after the activation by zymogens, the NaClO solution was able to block the activity of the activated form of MMP-9 (31% of reduction).

The treatment of THP-1 monocytes with the NaClO solution after activation with pooled WFs was able to reduce the gelatinolytic activity of total MMP-9 by a mean of 80% compared with the control condition. In particular, we observed a significant decrease in all three MMP forms, with a percentage of residual gelatinolytic activity of 32.9 ± 2.3 (*p* = 0.0004), 4.5 ± 4.5 (*p* = 0.0008), and 25.1 ± 11.4 (*p* = 0.0077) for MMP-9 at 225, 160, and 92 kDa, respectively.

#### 3.2.2. Peripheral Blood Serum

**Figure 3:**
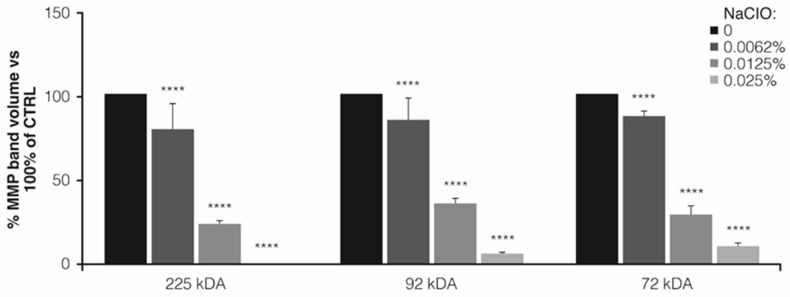
Detection of MMPs activity. Values are expressed as normalized peak area versus unstimulated THP-1 control cells, referred to as 100% (**** *p* < 0.0005).

The zymogram of peripheral blood serum showed three bands migrating at approximately 225 kDa (MMP-9 multimer form), 92 kDa (MMP-9 dimer form), and 72 kDa (monomer form). A significant reduction in the MMP-9 gelatinolytic activity was observed after the treatment with the NaClO solution. In particular, we observed that the treatment with 0.0062% of the NaClO solution decreased the gelatinolytic activity of all three forms compared with untreated controls (% of residual gelatinolytic activity vs. 100% of controls: 225 kDa, 79.9% ±9.0, *p* < 0.0001; 92 kDa, 84.6% ±8.9, *p* = 0.001; 87.6% ±2.5, *p* = 0.01, as shown in Figure 3). A more pronounced and significant decrease in gelatinolytic activity was observed by using 0.0125% and 0.025% of the NaClO solution (percentage of residual gelatinolytic activity vs. 100% of controls, −0.0125% NaClO solution: 225 kDa, 2.9% ±2.0, *p* < 0.0001; 92 kDa, 35.1% ±2.4, *p* < 0.0001; 29.5% ±2.9, *p* < 0.0001; 0.025% NaClO solution: 225 kDa, 0%, *p* < 0.0001; 92 kDa, 6.5% ±0.4, *p* < 0.0001; 10.9% ±0.6, *p* < 0.0001), as reported in Figure 3.

### 3.3. Quantitative Assay of MMPs Release

**Figure 4:**
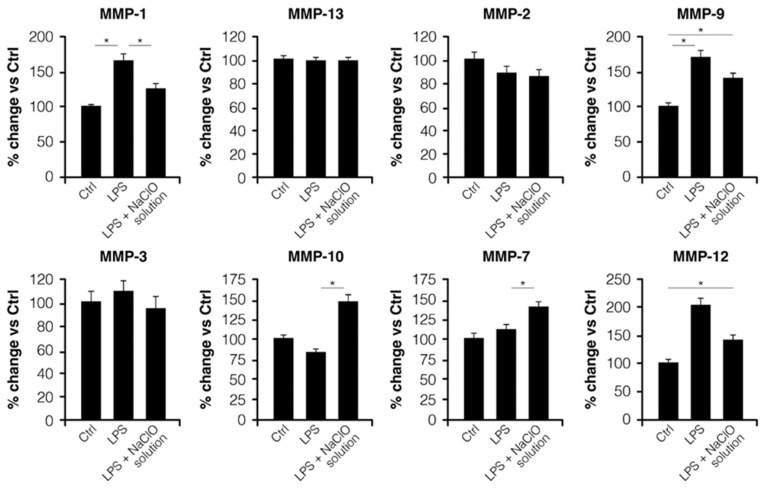
MMPs profile in LPS- and LPS + NaClO solution-stimulated THP-1 monocytes. Values are expressed as fold change versus unstimulated THP-1 control cells, referred to as 100% (* *p* < 0.05).

After the activation of THP-1 monocytes with LPS and the treatment with the NaClO solution, a significant reduction in MMP-1 (*p* < 0.05) and a slight reduction in MMP-9 and MMP-12 were reported compared with the LPS treatment alone (Figure 4); MMP-13, MMP-2, and MMP-3 showed a similar distribution (Figure 4).

### 3.4. Exploratory Scratch Wound Healing Assay

The wound surface area after LPS treatment was equal to 78.4% after 8 h and 66.5% after 24 h, compared with 39.0% and 38.9% of the control condition at the same time points, respectively. After the treatment with the NaClO solution, the wound surface area was 71.9% after 8 h and 51.5% after 24 h.

## 4. Discussion

Loss of functional balance among MMPs during the wound healing process is reported in literature evidence [1,7,8]. This imbalance can lead to an uncontrolled degradation of ECM elements, cytokines, growth factors, and their receptors, interfering with the healing process and contributing to the onset of chronic wounds [20]. According to literature data, fluid of chronic wounds is characterized by a significantly increased level of MMP-2 and MMP-9, supporting the hypothesis of an impaired proteases activity causing the degradation of the underlying ECM [1,9,10,11].

Our in vitro study suggests that a solution containing 0.05% NaClO is safe and able to reduce the gelatinolytic activity of MMP-9 and its activated form, released by activated macrophages. The downregulation of MMP-9 gelatinolytic activity was also observed in peripheral blood serum after the treatment with the NaClO solution.

The MMPs profile showed a significant reduction in MMP-1 release (*p* < 0.05) and a slight reduction of the release of MMP-9 and MMP-12 after the treatment with LPS and the NaClO solution, compared with the LPS treatment alone.

A slight improvement in wound healing was observed after macrophage activation with LPS and treatment with the NaClO solution.

Our observational data are supported by different mechanistic aspects of MMP inhibition by hypochlorite, as reported in literature in different human and animal models. The main mechanisms are related to: (1) the high alkaline pH of NaOCl, able to inhibit enzymatic activity [15]; (2) the reduction of inflammatory processes of NaOCl, modulating MMP expression by neutrophils [21,22], and (3) the well-known ability of NaOCl to alter the cysteine switch domain of some MMPs [23,24].

This study presents some limitations, such as the use of a monocytic cell line instead of the whole plasma, or the presence of the experimental data for only 8 types of MMPs out of 24 described in the literature. However, our results provide a further characterization of the activity of the NaClO solution which is useful to improve its use in clinical practice during wound care.

## 5. Conclusions

The results obtained suggest a possible ability of the NaClO solution to modulate the proteolytic pathways in the wound microenvironment, further characterizing its activity and improving its use in clinical practice during wound care.

## Data Availability

The datasets generated during and/or analyzed during the current study are available from the corresponding author upon reasonable request.
